# The Genetic Liability to Disability Retirement: A 30-Year Follow-Up Study of 24,000 Finnish Twins

**DOI:** 10.1371/journal.pone.0003402

**Published:** 2008-10-15

**Authors:** Karoliina Harkonmäki, Karri Silventoinen, Esko Levälahti, Janne Pitkäniemi, Antti Huunan-Seppälä, Timo Klaukka, Markku Koskenvuo, Jaakko Kaprio

**Affiliations:** 1 Department of Public Health, University of Helsinki, Helsinki, Finland; 2 The Local Government Pensions Institution, Helsinki, Finland; 3 Department of General Practice, University of Tampere, Tampere, Finland; 4 The Social Insurance Institution, Helsinki, Finland; 5 Department of Mental Health and Alcohol Research, National Public Health Institute, Helsinki, Finland; Roslin Institute, United Kingdom

## Abstract

**Background:**

No previous studies on the effect of genetic factors on the liability to disability retirement have been carried out. The main aim of this study was to investigate the contribution of genetic factors on disability retirement due to the most common medical causes, including depressive disorders.

**Methods:**

The study sample consisted of 24 043 participants (49.7% women) consisting of 11 186 complete same-sex twin pairs including 3519 monozygotic (MZ) and 7667dizygotic (DZ) pairs. Information on retirement events during 1.1.1975–31.12.2004, including disability pensions (DPs) with diagnoses, was obtained from the Finnish nationwide official pension registers. Correlations in liability for MZ and DZ twins and discrete time correlated frailty model were used to investigate the genetic liability to age at disability retirement.

**Results:**

The 30 year cumulative incidence of disability retirement was 20%. Under the best fitting genetic models, the heritability estimate for DPs due to any medical cause was 0.36 (95% CI 0.32–0.40), due to musculoskeletal disorders 0.37 (0.30–0.43), cardiovascular diseases 0.48 (0.39–0.57), mental disorders 0.42 (0.35–0.49) and all other reasons 0.24 (0.17–0.31). The effect of genetic factors decreased with increasing age of retirement. For DP due to depressive disorders, 28% of the variance was explained by environmental factors shared by family members (95% CI 21–36) and 58% of the variance by the age interval specific environmental factors (95% CI 44–71).

**Conclusions:**

A moderate genetic contribution to the variation of disability retirement due to any medical cause was found. The genetic effects appeared to be stronger at younger ages of disability retirement suggesting the increasing influence of environmental factors not shared with family members with increasing age. Familial aggregation in DPs due to depressive disorders was best explained by the common environmental factors and genetic factors were not needed to account for the pattern of familial aggregation.

## Introduction

Aging of the population, the tendency of employees to retire early and the costs of early exit from labor market, especially due to depression among employees have been major topics of policy and scientific debate during the last decades in all Western industrialized countries. In the 1970s and 1980s, the most common medical reasons for disability retirement were cardiovascular and musculoskeletal diseases. Interestingly, over the last two decades, depression has become a major cause of disability pensions and sickness absence even when no decisive increase in the overall prevalence of depression has been found [1]–[5]. The possible reasons for the increase in depression as a cause of work disability are presumably as complex as is the process that leads to work disability and early retirement. Disability retirement should be seen as a result of the interplay between individual and societal constraints and opportunities, including health and work related factors, occupational and other socioeconomic factors, current pension and employer policies, pension legislation, attitudes, values, expectations and desires towards work and retirement [6].

Diseases and medical reasons are clear predictors and also legal requirements of disability pension [6]. In the light of genetic liability behind common diseases [7]–[8], it is likely that genetic factors also contribute to liability of disability retirement, but no previous studies on the impact of genetic factors on the process of disability retirement have been carried out. Thus, extending the focus also to cover the contribution of genetic factors on disability retirement is justified. Based on the previous twin and family studies, a moderate to high estimates of heritability have been found for bipolar I disorder [9], schizophrenia [10], low back disorders [11], [12], cardio-vascular diseases [13], [14] and type 1 and type 2 diabetes [15]–[17]. Likewise twin and family studies have indicated that depression is at least partly a familial disorder, which mainly results from genetic influences [18]–[20]. The variance accounted for by known specific genes underlying these genetic factors is still at best modest, under 5–10%, despite recent rapid progress in identifying novel genes through genome-wide association studies [21].

To better understand the process and the risk factors of disability retirement due to any medical cause and especially due to depressive disorders, there is a clear need for longitudinal studies with both genetically and environmentally informative data. The main aim of this 30-year follow-up study with 24 043 Finnish twins was to investigate the contribution of genetic factors on disability retirement due to the most common medical causes and especially due to depressive disorders.

## Methods

### Data

The data were derived from the Finnish Twin Cohort Study [22]. Same-sex twins were ascertained from the Central Population Register, and a survey questionnaire was sent to them in 1975. Zygosity was determined in the questionnaire based on questions on similarity of physical appearance at school age classifying 93% of pairs as monozygotic (MZ) or dizygotic (DZ) twins. The validity of the self-reported zygosity was assessed by using genetic markers in a subsample of the cohort and found to be highly accurate. It was estimated that the probability of misclassification of a twin pair was 1.7% [23]. For the purposes of this study, those who were retired before January 1st 1975 were excluded (2702 twin pairs and 1671 twins from pairs with response only of one co-twin). In addition, those twin pairs known to have a bipolar disorder were excluded (26 twin pairs) [9]. Thus, the final sample for analysis consisted of 24 043 twin individuals including 1668 male MZ, 3931 male DZ, 1851 female MZ and 3736 female DZ complete twin pairs. Participants of this study were followed up from the beginning of the year 1975 to the date of disability retirement, the date when the person began to receive an old age pension, the date of death/emigration or to December 31^st^ 2004.

Information on retirement events during the follow-up period from January 1^st^ 1975 to December 31^st^ 2004, including disability pensions (DPs) with diagnoses based on the 8^th^, 9^th^ and 10^th^ revisions of the International Classification of Diseases (ICD), was obtained from the Finnish official pension registers: the Social Insurance Institution and the Finnish Centre for Pensions. In the Finnish pension system, the granting of illness based early retirement pension (disability pension or individual early retirement pension for 58–64 years old employees) requires medically confirmed illness, disease or injury which essentially restricts or prevents working. Disability pensions can also be granted to persons who have never worked (for example, a student in their early 20s). A long career and working conditions have been admitting criteria especially for the individual early retirement pension, which was first introduced in 1986 and in general abolished when the pension reform took effect gradually from the beginning of 2005. However, no essential social or pension legislative changes concerning the disability criteria occurred during the follow-up period of this study.

Diagnoses were encoded using ICD-8, ICD-9 and ICD-10, which were registered as part of pension decisions made by the Finnish insurance institutions, for depressive disorders (ICD-8: 2960, 300, ICD-9: 296 excluding bipolar disorders, 300, ICD-10: F32, F33, F34, F39), for all other mental disorders (except depressive disorders) (ICD8 & 9: 290–315, ICD section F), for cardiovascular diseases (ICD-10: I00-I99, corresponding sections of ICD-8/9), for musculoskeletal disorders (ICD-10: M00–M99, corresponding sections of ICD-8/9), and for other reasons. A medical certificate including the diagnoses made by the treating physicians is required in the disability pension application. In addition, the final diagnoses causing work disability are made by the insurance physicians working in the decision making pension institutions on the basis of the comprehensive medical information provided (including relevant medical records). Information on mortality and migration was derived from the Central Population Register of Finland. The record linkage was done by using the unique person numbers assigned to all Finnish citizens in the 1960s and currently a few days after birth.

### Statistical methods

Among all individuals, cumulative incidence rates of disability pensions (DPs) by gender and zygosity and incidence rates of DPs per 1000 person years by age and main diagnostic reasons were calculated. Pairwise analyses started by computing correlations in liability, which were calculated for disability retirement crosstabulating presence or absence of disability in twin pairs (i.e. both unaffected, one affected and both affected). Then, a series of frailty–models of the age at onset of all DPs, DPs due to depressive disorders, other mental disorders, musculoskeletal disorders, cardiovascular diseases and other reasons were fitted.

#### Discrete time frailty–model

**Figure 1:**
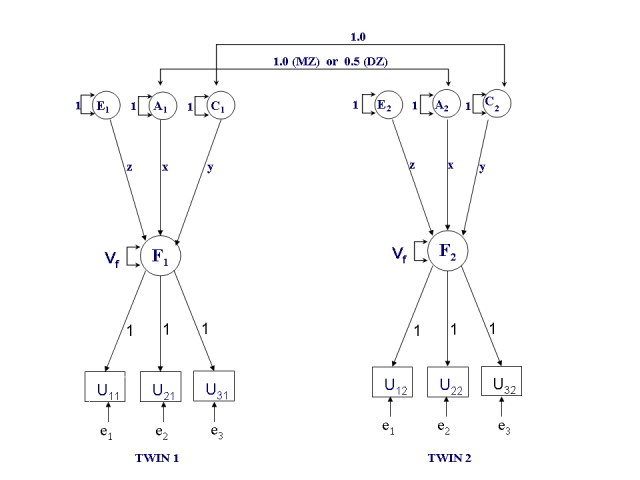
Structural equation model of the discrete time frailty for MZ and DZ twins at disability retirement, when age is splitted to three intervals (≤45, 46–55, 56–65). The estimates of the variance components are define as a) Heritability h^2^ = (x^2^)/(x^2^+y^2^+z^2^+1), b) Shared environment s^2^ = (y^2^)/(x^2^+y^2^+z^2^+1), c) Common unique environment u^2^ = (z^2^)/(x^2^+y^2^+z^2^+1) and d) Age interval specific unique environment ε_t_^2^ = 1/(x^2^+y^2^+z^2^+1). Correspondingly the path coefficients are defined as a) x = standard deviation (sd) of additive genetic effects, b) y = sd of shared environmental effects and c) z = sd of unique environmental effects. V_f_ represents the frailty variance common to age intervals.

We applied discrete time survival analysis (figure 1) with the probit link function to describe the relationship between hazard and latent factors [24]. Probit of the hazard function was modeled as a function of discretized age to be the outcome phenotype U_ti_ for time interval t (t = 1,2,3,4 or t = 1,2,3), corresponding age intervals ( ≤35, 36–45, 46–55, 56–65 or ≤45, 46–55, 56–65) and i (i = 1,2) indicating a twin. We used the following factor analysis model [25], [26]:
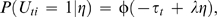
where φ( ) is the cumulative normal distribution function, τ_t_ is threshold for age interval t, λ matric of probit-regression parameters including path coefficients x, y, z and those path coefficients fixed to 1, and *η* is a continuous latent variable vector, including latent factors F_i_ (Dominance genetic effect), A_i_ (Additive genetic effect) C_i_ (Common environment effect) and E_i_(Unique environment effect). In expressing the model we follow the notation used by Muthén and Masyn [27].

Males and females were pooled together since number of concordant pairs was low for specific diagnostic reasons. Discretizing of event times was also based on number of concordant pairs: depending on diagnostic category, three or four age intervals were suitable number of intervals for twin analysis of event times. Maximum likelihood estimation was applied in Mplus statistical software [28]. The discrete time frailty–model for MZ and DZ twins when age is split into three intervals is shown in Figure 1.

The event of interest was disability retirement during the follow-up. Age at onset of event of interest was treated in discrete categories: the outcome phenotype U_ti_ for time interval t (t = 1,2,3,4 or t = 1,2,3), corresponding age intervals ( ≤35, 36–45, 46–55, 56–65 or ≤45, 46–55, 56–65) and i (i = 1,2) indicating a twin. The order of twins was at random. The indicator U_ti_ was defined as follows with three possibilities: twin i is at risk but did not experience the event in age interval t (value 0); twin i is at risk and experienced the event (value 1) and twin i is not at risk in age interval t and has been censored because of an occurrence of an event other than the event of interest (e.g. non-illness based early retirement event) or because of earlier occurrence of the event of interest (value 9). As part of the hazard model, a latent frailty variable (F_i_), common to age specific intervals, was defined to be a function of age interval specific event indicators. All factor loadings of the age intervals (e_t_) were assumed to be equal one as well as the variances of age interval specific frailties were fixed to one and assumed to be uncorrelated between twin pairs.

Variances and covariances of common frailties F_i_ were further specified according to the principles of classic twin modeling [29]. In the classical twin modeling genetic variation can be divided to additive genetic variation, which is the variance due to the additive allelic effects, and dominance genetic variation caused by interaction between alleles in the same locus, summed over all relevant loci. Epistatic effects, i.e. interaction between alleles in different loci, are assumed to be absent. Additive and dominance genetic effects have an expected correlation of one within MZ pairs and 0.5 and 0.25 within DZ pairs, respectively. Both MZ and DZ twins are assumed to share the same amount of environmental variation, which is partly shared by a twin pair (common environment C_i_), partly unique to each twin individual and age intervals (E_i_), and partly unique to each twin individual but specific to age intervals (frailties e_t_).

In our model formulation the effects of genetic and environmental factors on frailty were assumed to be constant between different age intervals. Based on the above assumptions, four sources of variation interpreted as latent and standardized variance components in a structural equation model can be identified: additive genetic (A), dominance genetic (D), common environment (C), and unique environment (E). Because our data includes only twins reared together, it does not allow modeling of genetic dominance and common environmental effects simultaneously, i.e. these effects are confounded.

The principle of parsimony was used, in other words we do not reject the complex (saturated) model (e.g., ACE) until evidence in support of a more simplex model (e.g., AE) requires us to abandon it. Chi-square goodness-of-fit (GFI) statistics, which are calculated by the difference between estimated −2 log-likelihood values of the saturated model (i.e. ACE model) and corresponding nested (restricted) model, were used to assess the fit of the model. This was done to compare the fit of the restricted models to the saturated model. Degrees of freedom of GFI-tests are calculated by difference of the degrees of these models. The superiority of restricted, hierarchically nested models was also assessed by Akaike information criterion (AIC; −2(log likelihood)+2(number of free parameters)). The model with the lowest value of AIC is considered the most parsimonious. The 95% confidence intervals for all parameters were estimated using bootstrapping.

## Results

### Disability retirement during the follow-up

During the follow-up, 4894 subjects (20% of the cohort) retired due to disability. Distribution of granted disability pensions (DPs) by diagnostic reasons is shown in Table 1. Males and participants over 45 years had a higher incidence of disability retirement than females and younger ones. For MZ males the cumulative incidence rate was 20.6% and for DZ males 23.0%; the corresponding incidence rates for females were MZ 16.7% and DZ 19.2%.

**Table 1:** Distribution of disability pensions by diagnostic reasons.

	Men		Women	
	MZ (n = 3538)	DZ (n = 8551)	MZ (n = 3907)	DZ (n = 8047)
**All Mental disorders**	4.7	5.1	5.1	5.5
**Depressive disorders**	2.3	2.4	3.1	3.5
**Other mental disorders**	2.9	3.4	2.7	2.8
**Musculoskeletal disorders**	6.8	8.0	6.7	7.9
**Cardiovascular diseases**	5.5	6.2	2.8	3.6
**All other reasons**	7.9	9.0	6.0	6.5
**All causes**	20.6	23.0	16.7	19.2

Cumulative incidence (%) among men and women for MZ and DZ individuals

**Figure 2:**
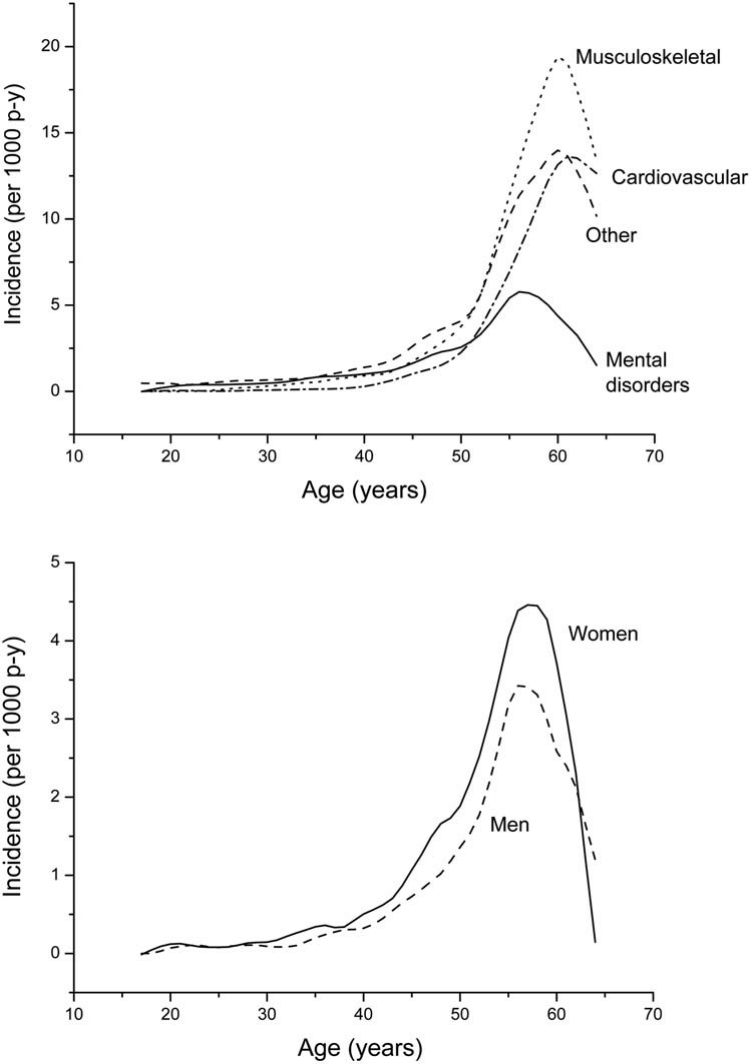
(2a) Incidence rates of disability pensions per 1000 person years according to diagnostic reasons (2b) Incidence rates of disability pensions because of depressive disorders per 1000 person years by gender.

Incidence rates of disability retirement increased with age, especially when disability pension was granted because of musculoskeletal and cardiovascular diseases. The age effect was clearly weaker if disability pension was granted due to mental disorders. After the age of 60 years, a lower incidence of disability retirement was observed in all main diagnostic groups (Figure 2a). Incidence rates of disability pensions due to depressive disorders per 1000 person years by age and gender are shown in Figure 2b. Especially after the age of 40, females had a higher incidence rate of disability retirement due to depressive disorders than males.

The number of concordant and discordant twin pairs for disability retirement in the main diagnostic groups is shown in Table 2. For all DPs the number of concordant MZ pairs (C+, both twins in a pair with disability pension) was 243, and the number of concordant DZ pairs was 521. For DPs caused by mental disorders the corresponding numbers of concordant pairs were 39 and 57, for DPs due to depressive disorders 11 and 18, for DPs due to musculoskeletal disorders 60 and 102 and for DPs due to cardiovascular diseases 28 and 52.

**Table 2:** The number of concordant and discordant twin pairs for disability pension

	Men						Women					
	MZ (n = 1668 pairs)			DZ (n = 3931 pairs)			MZ (n = 1851 pairs)			DZ (n = 3736 pairs)		
	C−	D	C+	C−	D	C+	C−	D	C+	C−	D	C+
**All disability pensions**	1143	393	132	2497	1134	300	1369	371	111	2538	977	221
**Mental disorders**	1530	122	16	3572	329	30	1689	139	23	3350	359	27
**Depressive disorders**	1596	68	4	3757	165	9	1746	98	7	3483	165	9
**Musculoskeletal disorders**	1475	163	30	3375	505	51	1649	171	30	3207	478	51
**Cardiovascular diseases**	1526	120	22	3513	384	34	1763	82	6	3498	220	18
**Other reason**	1444	194	30	3295	588	48	1652	181	17	3274	440	22

Note: C−: neither twin, D–discordant (one twin) and C+: concordant (both twins have disability pension)

### Effect of genetic factors on disability retirement

The correlations in liability for disability retirement in the main diagnostic groups are shown in Table 3. For all disability pensions, the correlation in liability was more than two times greater for monozygotic twin pairs (0.51) than for DZ pairs (0.18) in the youngest age group (≤45), in the middle aged (46–55 years) the MZ/DZ ratio was less than 1.5 (0.33 vs. 0.23) and only marginally greater in MZ (0.28) than DZ pairs (0.24) in the oldest age group (56–65 years). For mental disorders a consistent MZ/DZ difference was seen in all age-groups, while the MZ/DZ difference was small in the oldest age group for musculoskeletal disorders.

**Table 3:** Correlations in liability (95% CIs) for disability retirement in the main diagnostic groups.

	≤45		46–55		56–65	
	MZ	DZ	MZ	DZ	MZ	DZ
All disability pensions*	0.51 (0.39 , 0.61)	0.18 (0.09 , 0.28)	0.33 (0.23 , 0.42)	0.23 (0.16 , 0.29)	0.28 (0.16 , 0.39)	0.24 (0.17 , 0.32)
**Mental disorders**	0.67 (0.54 , 0.77)	0.26 (0.11 , 0.39)	0.37 (0.17 , 0.54)	0.27 (0.13 , 0.41)	0.19 (−0.21 , 0.54)	0.03 (−0.22 , 0.27)
**Depressive disorders**	0.56 (0.30 , 0.74)	0.15 (−0.17 , 0.44)	0.32 (0.06 , 0.55)	0.14 (−0.07 , 0.35)	0.31 (−0.13 , 0.65)	0.14 (−0.13 , 0.40)
**Musculoskeletal disorders**	0.57 (0.31 , 0.75)	0.34 (0.14 , 0.51)	0.34 (0.16 , 0.49)	0.16 (0.04 , 0.28)	0.28 (0.11 , 0.44)	0.25 (0.14 , 0.35)
**Cardiovascular diseases**	0.39 (−0.10 , 0.89)	0.45 (0.15 , 0.68)	0.38 (0.13 , 0.58)	0.24 (0.09 , 0.38)	0.37 (0.17 , 0.54)	0.27 (0.14 , 0.40)
**Other reason**	0.21 (−0.08 , 0.47)	0.18 (0.01 , 0.34)	0.38 (0.23 , 0.52)	0.22 (0.11 , 0.33)	0.14 (−0.08 , 0.35)	0.10 (−0.04 , 0.24)

Table 4 shows the results of age at onset for all DPs and cause specific DPs due to mental disorders, musculoskeletal disorders, cardiovascular diseases and for other reasons. The model of genetic and specific environmental factors (AE) was the best fitting model in all DPs and in the main diagnostic groups. The heritability estimate for all DPs was 0.36 (95% CI 0.32–0.40) and for main diagnostic categories ranged from 0.24 for all other reasons to 0.48 for cardiovascular diseases. Shared environmental effects could be dropped from all models on these outcomes.

**Table 4:** Estimates (95% CIs) of variance components based on the AE model for age at onset of disability pensions.

	h^2^	u^2^	ε^2^
**All disability pensions**	0.36 (0.32 , 0.40)	0.12 (0.07 , 0.17)	0.52 (0.48 , 0.57)
**Mental disorders**	0.42 (0.35 , 0.49)	0.04 (0.01 , 0.07)	0.54 (0.48 , 0.60)
**Musculoskeletal disorders**	0.37 (0.30 , 0.43)	0.00 (0.00 , 0.00)	0.63 (0.57 , 0.70)
**Cardiovascular diseases**	0.48 (0.39 , 0.57)	0.27 (0.17 , 0.38)	0.25 (0.18 , 0.32)
**Other reason**	0.24 (0.17 , 0.31)	0.04 (−0.01 , 0.09)	0.72 (0.65 , 0.79)

The model fitting results for DPs due to depressive disorders are shown in Table 5. Model-fitting started with an ACE model, in which the individual variance components (aside from age-specific environmental effects) were each non-significant. Dropping C from the model resulted in a worse fitting model (p = 0.035 for chi-square change; Δχ^2^ = 4.430, Δdf = 1); in the resulting AE model a significant genetic component could be observed. In contrast, additive genetic effects could be dropped without a significant worsening of the model fit (p = 1.0). Hence, the best fitting model for DPs due to depressive disorders was the model with common and specific environmental components (CE). A pure E model fitted very poorly, indicating that there was significant familial aggregation in the liability to DP from depressive disorders. In the CE model, 28% of the variance was explained by the common environmental factors (95% CI 21–36). The age interval specific unique environment explained 58% of the variance in DPs due to depressive disorders (95% CI 44–71).

**Table 5:** Estimates (95% CIs) of variance components and the goodness-of-fit statistics of series of models for disability pensions due to depressive disorders.

	Standardized estimates of the variance components				Model fit compared to full ACE+ε−model			
	h^2^	s^2^	u^2^	ε^2^	χ^2^	df	p	AIC
**ACE+**ε	0.06 (−0.30 , 0.42)	0.22 (−0.03 , 0.47)	0.06 (0.00 , 0.11)	0.66 (0.55 , 0.77)	–	–	–	–
**AE+**ε	0.35 (0.25 , 0.46)	–	0.12 (0.03 , 0.21)	0.53 (0.43 , 0.63)	4.430	1	0.035	2.43
**CE+**ε	–	0.28 (0.21 , 0.36)	0.14 (0.02 , 0.26)	0.58 (0.44 , 0.71)	0.000	1	1	−2.00
**E+**ε	–	–	0.20 (0.11 , 0.28)	0.80 (0.72 , 0.89)	44.51	2	<0.001	40.51

## Discussion

This study on the effect of genetic factors on disability retirement due to the most common medical causes and especially due to depressive disorders was based on 24 043 Finnish twins, of whom 4894 retired due to disability during 30 years of follow-up. To our knowledge, this is the first study that has examined the influence of genetic factors on disability retirement. Thus, the results of this study provide novel information on the factors affecting disability retirement during the life course. The moderate genetic contribution to the variation of all granted DPs (heritability estimate of 36%) and other mental disorders than depressive disorders was found (42%) indicating the importance of both genetic factors and different environmental exposures. When interpreting the results of this study, it should be understood that heritability is not informative about possible gene-environment interactions and heritability is a population-specific estimate observed for the population at hand. If an intervention is undertaken, even a trait or disease with a high heritability can be changed.

The effect of genetic factors mostly decreased with increasing age. Thus, most of the variance in disability retirement due to any medical cause was explained by the environmental factors not shared by a twin pair. The highest heritability estimate was found in DPs due to cardiovascular diseases (48%). The model with common and specific environmental factors showed the best fit in DPs due to depressive disorders indicating also the important impact of the shared early family environmental factors.

Interestingly, the incidence rates of DPs in DZ twins were consistently higher than the incidence rates in MZ twins. It may be due to various causes, including greater interactions between cotwins in MZ pairs relevant for the etiology of DPs. MZ twins have greater social interaction with each other, and this increased social support might make it likely that MZ twins delay seeking DP compared to DZ twins. Other mechanisms are possible and this finding should be further evaluated.

Since no previous studies on the effects of both genetic and environmental factors on disability retirement have been carried out before, the compatibility of the results of this study to the previous ones cannot be evaluated. However, the results concerning the effect of genetic factors on DPs due to cardiovascular diseases and musculoskeletal disorders resembled the findings of the previous twin studies. In a recent study of Battié et al. [11], the heritability estimates for various measures of low back pain ranged from 30% to 46% among middle-aged adults. Substantial heritability estimates have been found for neck pain [30] and for rheumatoid arthritis [31] and moderate heritability estimates have been shown for coronary heart disease [14], [32], stroke [33] and hypertension [13]. The role of genetic factors both in musculoskeletal disorders and in cardiovascular diseases (CHD) has been found to be greater at younger adults than in older adults [34], [35], [30], [32]. Of note is that our analysis focuses on working-age persons.

Previous twin and family studies have reported high heritability estimates for schizophrenia and bipolar disorders, which have also been seen in this same cohort [9], [10]. In the present analysis, the heritability estimate for DPs due to mental disorders was 42%, which is probably due to the etiological heterogeneity of the broad class of mental disorders as well as due to specific factors leading to disability pension among those with a diagnosis. Only a part of those with a diagnosed severe mental disorder are incapacitated and retire early due to disability.

Depression has been found to be at least partly a familial disorder, which mainly results from genetic influences and only minor importance has been given to shared environment [20], [36]. In most twin studies, the heritability of liability to major depression has ranged from 30% to 40% [20], [37], [38], which is consistent with the point estimate from the AE-model in our analysis. However in this study, the familial aggregation in DPs due to depressive disorders was best explained by the shared environmental factors. For DPs due to depressive disorders, the model with genetic factors fitted the data somewhat worse than the model without genetic factors. In the genetic model, the estimate of heritability for DPs due to depressive disorders was 35%; twin studies have relatively poor power to decisively distinguish between genetic and non-genetic familial effects. Despite a very large initial sample size and thirty years of follow-up, there were only 11 MZ and 18 DZ pairs concordant for disability pension due to depressive disorders, which limited the possibilities for more detailed analysis by sex or age. It is possible that the comprehensive prospective follow-up of cases rather than retrospective, interview-based assessment of lifetime depression used in most earlier studies may have contributed to the difference in findings. It is also possible that the cases of depression for which disability pension is granted represent the more severe and less treatable cases in the population. Thus, our results cannot be directly compared with earlier results using other case ascertainment methods and reasons for those differences need to be explored more carefully, for example by examining the predictors and correlates of disability pension due to depression.

This finding on the impact of shared early family environment is, however, interesting. The results of a recent population based prospective study [39] showed that the risk of disability retirement increased in a dose-response manner with increasing number of childhood adversities. Recent research evidence indicates that the vulnerability to depression is influenced by early life experiences and relationships also in Finland [40], [41] and the importance of the interaction effects between genes and early childhood environment on the development of depression has currently been emphasized [37], [42], [43].

Previous studies have reported that low socioeconomic status, poor somatic health, stressful life-events, interpersonal conflicts, low social support, negative childhood experiences, stressful psychosocial working conditions and health related risk behaviour are associated with increased risk of depression [44]–[50] [40], [41], and a higher risk of disability retirement has been reported to be associated with these same risk factors [51]–[55] [39].

The strength of this study includes the large study sample with high initial response rate (89%). There was a considerable long follow-up period and reliable, comprehensive register based information on disability retirement. In this study no personal contact to study subjects was required and virtually all subjects could be traced through the population and medical registers; loss to follow-up was therefore minimal or none. The youngest subjects were aged 18 at entry covering thus the entire working aged population. In addition, the advanced and complex statistical methods used in this study are considered as strength of this study. A possible weakness of the study is the changes of the International Classification of Diseases (the 8^th^, 9^th^ and 10^th^ revisions of the ICD), which has affected changes in the diagnostic criteria of many diseases, but particularly mental disorders and depression. Case definition was based on multiple medical records and review by experts. However, despite the possible heterogeneity in the process of becoming disabled especially due to mental disorders during the working life period, our twin study shows that genetic factors appear to be important for becoming disabled overall as well as for the main diagnostic categories.

The results of this study on the contribution of genetic factors to disability retirement, and insights from further studies on the complex associations between genetic and environmental factors on the process of disability retirement during the life course, provide better focused tools for planning strategies to prevent work incapacity and early retirement among employees. For example, analyzing twin pairs discordant for different work loading factors offer a powerful tool to analyze whether these factors affect directly to early retirement due to disability or whether these associations are because of correlated factors. In addition, further studies are needed to examine the possible gene-environment interactions, gene-environment correlations and other complex models of the relationship of genetic and environmental factors. Understanding of the influence of both the genetic and different environmental factors during the life course to the process of disability retirement will provide better focused tools for planning strategies to prevent work incapacity and early retirement among employees.
